# Translating time: Challenges, progress, and future directions

**DOI:** 10.1016/j.brainresbull.2025.111212

**Published:** 2025-01-15

**Authors:** Christine J. Charvet, Alexandra A. de Sousa, Tatianna Vassilopoulos

**Affiliations:** Department of Anatomy, Physiology & Pharmacology, College of Veterinary Medicine, Auburn University, Auburn, AL 36849, USA

**Keywords:** Translating Time, Human, Chimpanzee, Mouse, Evolution

## Abstract

Mice are the dominant model system to study human health and disease. Yet, there is a pressing need to use diverse model systems to address long-standing issues in biomedical sciences. Mice do not spontaneously recapitulate many of the diseases we seek to study. Accordingly, the relevance of studying mice to understand human disease is limited. We discuss examples associated with limitations of the mouse model, and how the inclusion of a richer array of model systems can help address long standing issues in biomedical sciences. We also discuss a tool called Translating Time, an online resource (www.translatingtime.org) that equates corresponding ages across model systems and humans. The translating time resource can be used to bridge the gap across species and make predictions when data are sparse or unavailable as is the case for human fetal development. Moreover, the Translating Time tool can map findings across species, make inferences about the evolution of shared neuropathologies, and inform the optimal model system for studying human biology in health and in disease. Resources such as these can be utilized to integrate information across diverse model systems to improve the study of human biology in health and disease.

## 1. Introduction

Neuroscience has a rich history of studying diverse groups of species to uncover fundamental principles of brain structure and function. Species as wide ranging as zebra finches, bats, cats, squids, rhesus macaques, and voles, have all provided insights into the neural basis sensations and behaviors in health and in disease (Moss and Sinha, 2004; Striedter, 2005; Keifer and Summers, 2016; Buffenstein et al., 2022; Farris, 2020). For example, studying giant axons in squids gave insight into the action potential, which is a fundamental mechanism for neuronal communication (Striedter, 2016). Some animals (e.g., cats, dogs) spontaneously develop diseases like cognitive dysfunction syndrome making them valuable model systems of human aging. Each species offers complimentary insights into the human brain because different model systems possess unique advantages to advance our understanding of human biology. Some animals, such as mice, mature and age quickly compared with humans, and so can be studied in a timely manner (Bayer et al., 1993; Brenowitz and Zakon, 2015). Nonhuman primates (e.g., macaques, marmosets) are useful model systems to track human biology in health and disease due to their close phylogenetic proximity to humans (Mitchell and Leopold, 2015; Passingham, 2009; Phillips et al., 2014; Rigby Dames et al., 2023). Yet, other systems display unique sensory and cognitive repertoires (e.g., echolocation, social bond formation). These specializations can be used to investigate how genetic and environmental variation shapes brain and behavior (Kuc, 1994; Krubitzer et al., 1993, 1995; Ache and Young, 2005; Catania, 2005). Diverse model systems have provided complimentary information crucial for studying problems in biomedical sciences.

In recent decades, mice have become the predominant model system in biomedical sciences, partly because mice develop quickly, breed rapidly, and are amenable to genetic manipulations (de Sousa et al., 2023). Different model systems are still used in science today (Clancy et al., 2001, 2007), however, the use of these model systems (e.g., rabbits, guinea pigs, and gerbils) have diminished considerably over time (Supplementary Fig. 1). Focusing on a restricted number of model systems can limit perspectives in biomedical sciences (Brenowitz and Zakon, 2015; Gould et al., 2015; Eaton and Wishart, 2017). Notably, many complex diseases, including Alzheimer’s disease, Parkinson’s disease, and autism spectrum disorder are not fully recapitulated in mice (Marx et al., 2021). To address this issue, mice are genetically manipulated to artificially recreate diseases. However, these genetically modified mice do not represent the full spectrum of human diseases. Findings obtained from mice do not necessarily translate to humans (Brenowitz and Zakon, 2015). Other species may spontaneously develop diseases found in humans, but relatively less effort has focused on studying these animals, even though such investigations might be advantageous and translatable to humans. Therefore, it would be beneficial to diversify the species and approaches utilized in biomedical sciences (Box 1).

Previously, we developed an easily accessible website that enables investigators to find corresponding ages across species, which allows researchers to translate findings across model systems and humans (www.translatingtime.org). The comprehensive ‘Translating Time’ resource offers researchers a standardized framework to compare developmental processes across mammals and to predict the timeline of biological processes as needed (Clancy et al., 2001; Charvet et al., 2023; Rakic, 1974). Across mammals, there is a particular sequence with which developmental programs typically unfold (Catania et al., 2005; Clancy et al., 2001; Rakic, 1974; Atlas, 2013). First, we discuss how we find corresponding ages across species using Translating Time. Then, we discuss current challenges we are addressing with the Translating Time framework, including finding appropriate models of human development and aging, and how our most common model system, the laboratory mouse, is limited in resolving challenges in biomedical sciences. Finally, we discuss how efforts to improve the health of humans and animals (i.e., a One Health framework) are aligned with addressing these challenges (Atlas, 2013; Natterson-Horowitz, 2015). We discuss how more efficient scientific organization, including large-scale collaborations, open science, and others, presents exciting approaches to achieve the ambitious goal of integrating comparative research in biomedical sciences.

## 2. Section I: challenges

### 2.1. Mice as model systems are limited

Laboratory mice were chosen as a reference model organism mainly because they develop quickly and are easy to breed in the laboratory. Yet, the study of laboratory mice is unlikely to be sufficient to tackle human health for several reasons. 1) First mice do not share many biological features with humans. For example, laboratory mice lack features of pathology found in humans with Alzheimer’s disease (AD), that is, they do not naturally express Amyloid beta (Aβ) plaques nor Tau tangles (de Sousa et al., 2023). These features naturally occur in a broad range of mammalian species so other model systems may be suitable. Perhaps, mice do not live long enough to recapitulate all the stages of the human lifespan and their concomitant diseases (Cottam et al., 2024). Mice have been chosen as laboratory mammals specifically because they have accelerated lifespans and reproduce quickly, which are features that humans lack. The distinct life histories of mice versus humans may limit their suitability for studying the most comprehensive aspect of human biology: aging, where the cumulative effects of diverse biological processes converge over a long lifespan. 2) Second, findings from mice may not easily translate to other species because they have evolved to adapt to laboratory settings, which is a very different environment from those of humans. Studying many species is needed to ensure findings from model systems relate to humans.

We could study health in ways that enhance human health while also benefiting other species, including companion animals that live with us, livestock we depend on for food, and endangered animals whose conservation supports biodiversity. 3) Finally, studying mice does little to promote and protect the environment, which we urgently must sustain. Mice were selected as model species precisely because they are so resilient within anthropocene environments. Accordingly, studying mice that are bred for laboratories does not provide the knowledge we need to support those species that are most vulnerable because lessons learned from laboratory mice are not necessarily relevant to animals in complex environments and ecosystems. Panksepp (2005) suggested that we should examine animal models in relevant environments to study their natural behaviors. For virtually everything we need to understand about human health, animal health, and the environment, the conditions met by laboratory mice are not directly relevant to those of humans or companion animals. It remains an open question whether laboratory mice are representative of wild mice. Animals that were bred for those conditions are likely to be poor models for diseases, which arise from the interaction of genotypes and environments.

Complex diseases arise from the cumulative effects of genetics, lifestyle choices, and environmental exposures. Unlike simple genetic disorders, which can be traced to mutations in a single gene, complex diseases involve a network of genes and their interactions with the environment, making their study and treatment significantly more challenging than simple diseases. Mice are frequently used to study complex diseases even though they are raised in laboratory settings, which differ substantially from human environemnt, making them poorly suited for studying complex diseases. Examples of complex diseases include diabetes, cardiovascular disease, cancer, dementia, cataracts, and glaucoma. The complexity of these diseases often leads to variation in how these diseases manifest and how individuals respond to treatment. These challenges have led to the development of personalized approaches in both research and clinical settings. Considering a breath of species, and especially those that naturally recapitulate diseases has much potential to inform human medicine. Approaches that integrate information from animals in their natural environment and those that are focused on enhancing animal and human health (i.e., One Health) are exciting yet untapped opportunities. We have grown a resource that integrates findings across diverse model systems so that information gained across model systems can be collectively used to inform human medicine.

## 3. Section II: progress

**Fig. 1.**
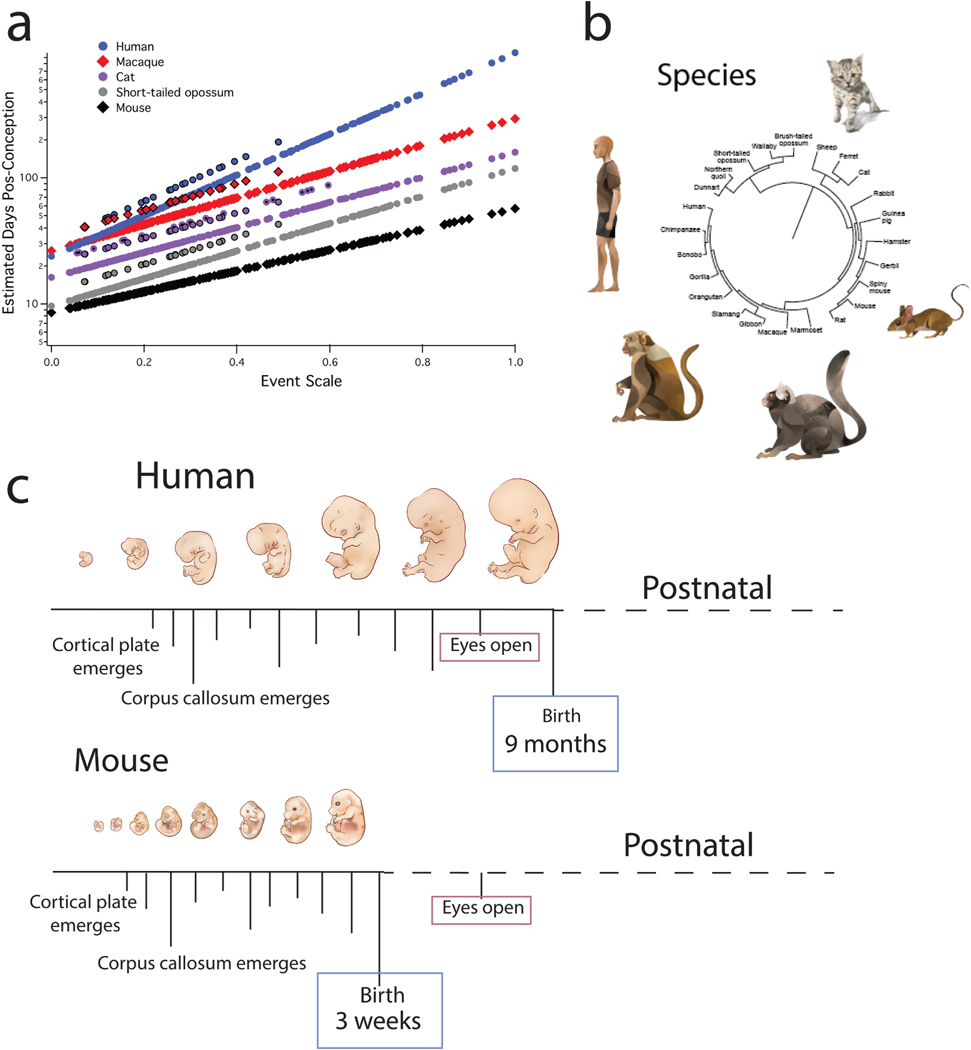
Overview of methods used to translate ages across species. (a) Predicted time points are plotted against an event scale, which is an ordering of time points. This model is used to find corresponding ages across species (www.translatingtime.org). (b) The Translating Time resource includes a broad range of species. (c) Examples of time points (e.g., cortical plate emerges) used for cross-species age alignments are shown for humans and mice. (b) The phylogeny is generated with phyloT. (a) This graph is modified from Workman et al. (2013) and is from Charvet and Finlay (2018).

**Fig. 2.**
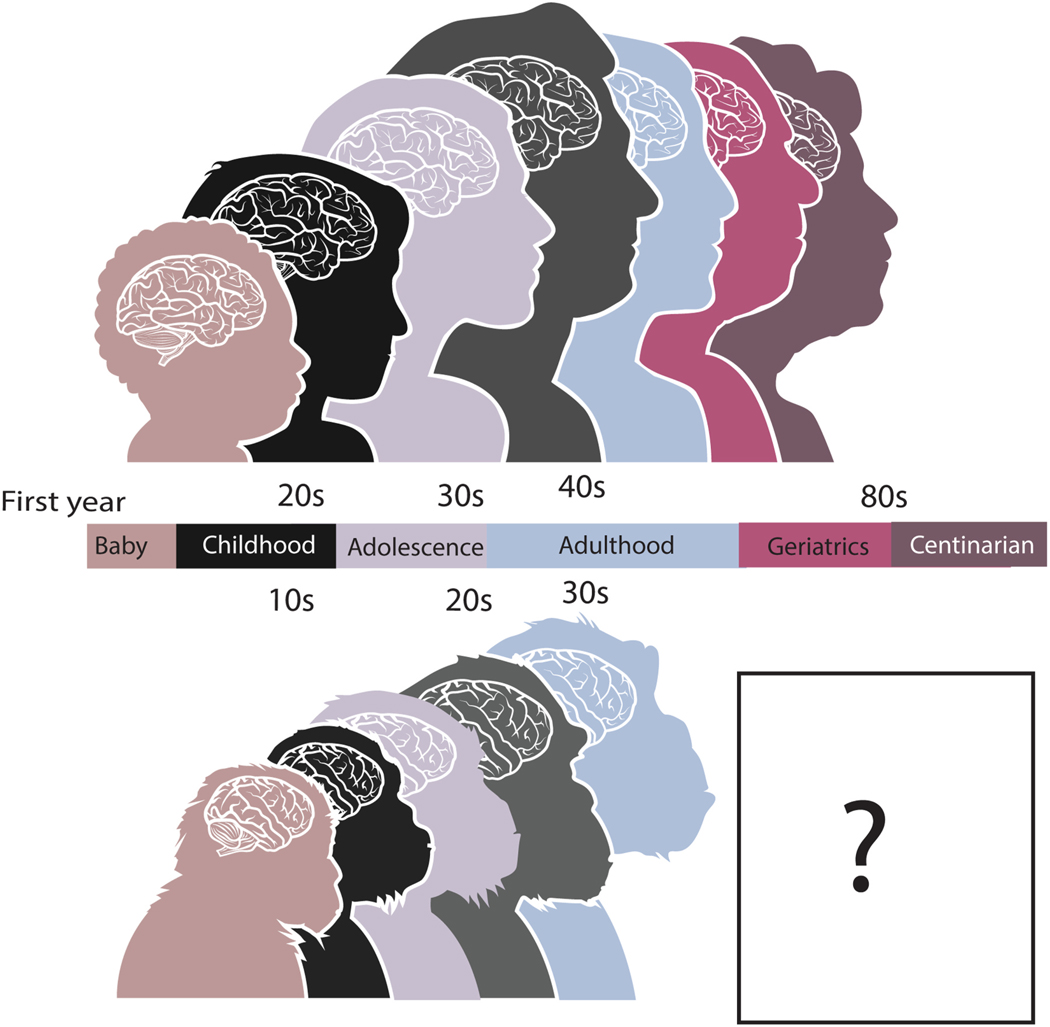
We used time points to find corresponding ages across humans and chimpanzees (Raff and Wray, 1989). Humans are surprisingly similar to chimpanzees though humans do take slightly longer to develop, and age compared with chimpanzees, but chimpanzees rarely live to the equivalent of a 70-year-old human and beyond so that there is a phase of life with no clear correspondance between humans and chimpanzees.

Diseases emerge at different phases of our life. Therefore, it is imperative to study model systems that closely align with the corresponding human age. Our Translating Time resource allows researchers to easily equate ages across humans and model systems, enabling researchers to integrate findings across model systems, and to harness available information across model systems to inform human health. The current database for the “Translating Time” model translates equivalent ages in humans and model organisms (www.translatingtime.org ). This resource is the product of collaborative efforts across multiple individuals and decades (Fig. 1). Initially, Finlay and Darlington (1995), extrapolated when different neuronal populations are born across multiple brain regions, and used these data to generate cross-species age alignments (Clancy et al., 2001). Subsequent work led by Drs. Clancy and Finlay increased the breadth of developmental processes (e.g., synaptogenesis, cell death, myelination), and more coverage across species (i.e., in total, 271 time points), which enabled cross-species age alignments up to 2 years of age in humans and their equivalent in other species (Clancy et al., 2001, 2007). The inclusion of temporal variation in transcription, anatomy, and behavior increased the dataset to 573 time points across humans and great apes. This approach permitted finding corresponding ages across the lifespan of humans and some primate species (Fig. 2; (Clancy et al., 2001; Charvet et al., 2023; Rosati et al., 2023). The specific Translating Time model has evolved over the years but has continuously relied on the collation of time points collected across a range of ages and species.

We first generate an event scale, which is a relative ordering of time points with values varying from 0 to 1 with early events assigned a score close to 0 and late events assigned a score close to 1 (Clancy et al., 2007; Charvet et al., 2023). Some time points are available in a subset of species, resulting in missing data. In creating the event scale, we impute data that are missing so that we can generate an ordering of time points averaged across all studied species. A model is then fit to the original dataset (un-imputed data) and the event scale (which includes imputed data). The y-axis represents the age of occurrence of each time point in each species, expressed in log-transformed days after conception, while the the event scale is plotted on the x axis (Fig. 1). The model can find corresponding ages for any of the studied animals. The model in forms how the pace of development varies across species. For example, time points in humans and macaques are similar early on just before neurogenesis (e.g., around embryonic day 40), but corresponding time points occur much later in humans than they do in macaques at postnatal ages (Fig. 1). Examples such as these show that corresponding biological pathways become progressively divergent with age in slow-maturing versus fast-maturing species. The model can predict age based on known biological events and predict the age at which specific biological processes should occur. Time points are extracted from different methods like neuroimaging, gene expression, morphometric changes, and behavior as well as neurodevelopmental data collected previously. These include neurogenesis of different cell types assayed from birth-dating techniques (Rakic, 1974; Finlay and Darlington, 1995). The collation of time points from multiple metrics provides a comprehensive approach to translate ages across species. The predictive power of the model is especially valuable when biological processes cannot be studied directly as is the case for many human biological processes.

**Fig. 3.**
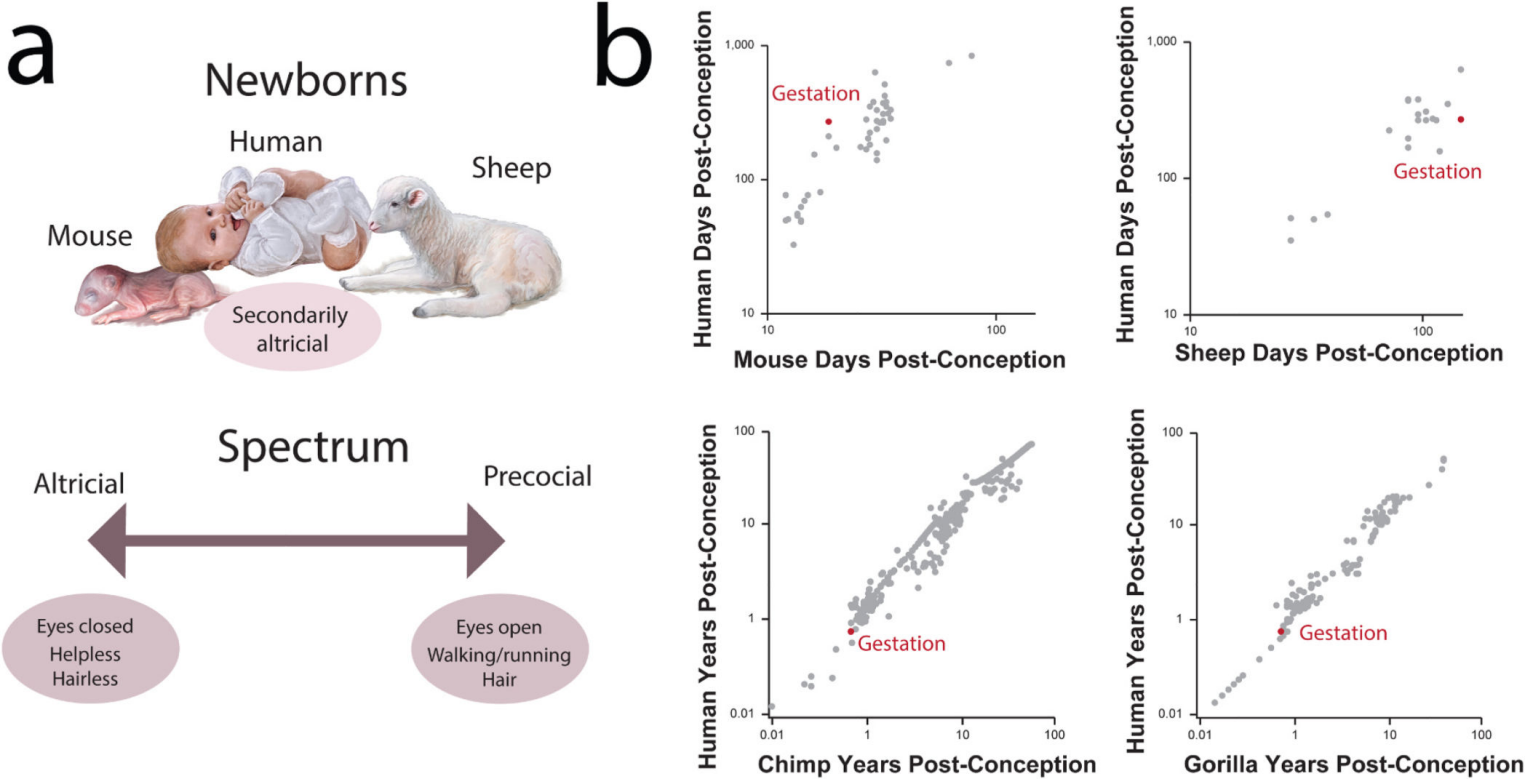
Species vary widely in their maturity state at birth. (a) Some species are altricial (e.g., mouse) in that they are born in a relatively immature state. Other species (e.g., sheep) are born in a relatively mature state. Still other species, such as humans, are intermediate in their maturity state at birth. Humans are secondarily altricial. (b) Scatterplots of time points in select species. These data are from Workman et al., 2013 and Charvet et al., 2023.

Many biological processes during fetal development are abrupt and occur within a span of hours or days, whereas other changes vary more gradually at later stages of life. The onset, peak, and termination of neuron birth and the emergence of neuroanatomical structures can be determined within a few days in many studied model systems. A time point may be based on a sample of individuals or a single individual. As development progresses into aging, biological and behavioral changes span longer time scales and may occur over the span of months or years. Age alignments can be extracted from these gradual changes, provided they occur similarly in the studied species. Time points from both gradual and abrupt changes can be used to generate cross-species age alignments, and we have integrated such changes to find corresponding ages across the lifespan of different species (Charvet et al., 2023; Cottam et al., 2024; Charvet, 2021). For example, we applied this approach to translate ages across the lifespan of humans and great apes (Charvet et al., 2023; Charvet, 2021). Humans and great apes are expected to share many biological and behavioral changes because of their close phylogenetic proximity. One notable finding to emerge from this work is that humans do take a little bit longer to mature and age compared with chimpanzees Accordingly, chimpanzees and humans progress through similar life stages, including childhood, adolescence, adulthood, and old age (Fig. 3); 24, 32. Yet, very few chimpanzees live past their 40 s (the equivalent of 50 s in humans) so that lifespan is on average much shorter than in humans. The relatively extended lifespan of humans means that humans may experience certain biological changes at late stages of life that are not evident in chimpanzees. This extended lifespan should increase the risk of developing certain age-related diseases (e.g., Alzheimer’s disease, cataracts) in humans (Chen et al., 2013; Rosati et al., 2023; Jurmain, 1989, 2000; Kumar et al., 2017).

The Translating Time resource is based on hundreds of time points. As such, the resource can be used to detect changes in the timing or rate of development in a taxonomic group. Extensions or contractions in the duration of specific biological processes may accentuate or reduce the size, shape, and function of body parts (Gould, 1977; McNamara, 1982; Raff and Wray, 1989). Gould’s view of heterochrony focused on relative rates of growth, although there are several types of heterochronies across vertebrates. Heterochronies can arise by varying the relative timing of process commencement, cessation, or changes in growth rate. Heterochronies are also observed across sexes and are linked to sexual dimorphism (Kumar et al., 2017; Gould, 1977; McNamara, 1982; Raff and Wray, 1989; McNamara, 2012). There are many heterochronies, which account for cross-species variation in locomotor capabilities, skull shape, and brain size in adulthood. A heterochrony is defined here as a time point that deviates from most others. Collecting observations from diverse biological processes is essential to identify relative modifications in the timetable of biological or behavioral processes. We previously tested whether the inclusion of factors for certain biological processes (e. g., cortical neurogenesis, retinal neurogenesis) accounts for a significant percentage of the variance in the model. If the inclusion of these factors in the model does account for a significant percentage of the variance, we define these processes as heterochronies. We have previously found that cortical neurogenesis is relatively extended in primates compared with rodents, and this developmental change accounts for the expansion in cortical neuron numbers, especially in the primate visual cortex (Bayer et al., 1993; Clancy et al., 2007; Rakic, 1974; Charvet et al., 2017; Florio and Huttner, 2014; Finlay et al., 1998). These studies show that relative changes in the timetable of neurogenesis leads to major modifications in the neural circuit responsible for primates’ ability to detect visual information. We have found multiple instances of heterochronies in different taxonomic groups.

### 3.1. Translating time to predict biological processes

The Translating Time resource is useful to predict the pace of biological pathways because it can guide the optimal age an animal should be studied. We provide an example focused on the temporal progression of human hippocampal neurogenesis and show how information from model systems can be used to make predictions in humans.

#### 3.1.1. Translating time across multiple species: human hippocampal neurogenesis

The use of multiple model systems can address questions that are difficult to resolve in humans. For example, the existence of human hippocampal neurogenesis has been difficult to conclusively address in young and aged humans because human tissue is rarely accessible for study, and because the amount of hippocampal neurogenesis is at best low in young and aged adults (Boldrini et al., 2018; Lee and Thuret, 2018; Sorrells et al., 2018). In cases such as these, cross-species age alignments can be used to test whether the temporal progression of hippocampal neurogenesis in humans follows the temporal pattern found in model systems (Charvet and Finlay, 2018; Amrein et al., 2011, 2015). Studying one model system risks assuming that the model system has evolved a derived trait, and we might assume the extrapolation of derived traits is the norm that should be applied to humans. Identifying conserved patterns in the temporal decline of hippocampal neurogenesis by studying multiple species is very useful because conserved processes are most applicable to humans.

**Fig. 4.**
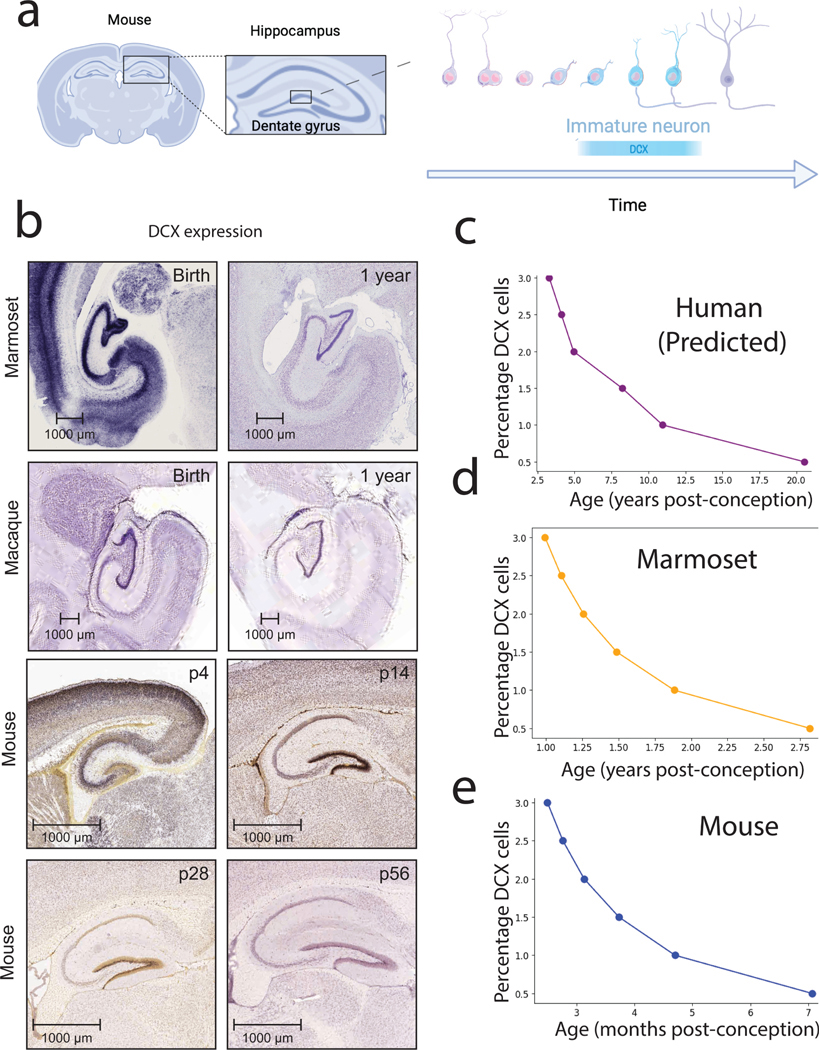
We can use information on hippocampal neurogenesis from model systems to predict the temporal pattern in humans. (a) In the dentate gyrus, newly born neurons are generated. Doublecortin expression (DCX) can be used to quantify the extent of hippocampal neurogenesis. (b) DCX expression in marmosets, macaques, and mice brains is high shortly after birth, and subsequently declines postnatally. DCX expression is still observed after 1 year of age in macaques as in marmosets, but it is observed at very low levels thereafter. The relative number of DCX+ numbers can be used to assess the extent of hippocampal neurogenesis. (c) Predicted time course in DCX in humans based on those of other species, including marmosets (d) and mice (e). (b) In situ hybridization data are from the in situ hybridization Allen Brain Institute (http://mouse.brain-map.org).

During prenatal development, high rates of hippocampal neurogenesis are found in diverse species, including mice, macaques, and marmosets, but levels decline through postnatal and juvenile development. Such a decline in hippocampal neurogenesis is a common trend across studied mammals. Various methods can be used to study neurogenesis directly or indirectly, but the gold standard methods are birth-dating experiments, which involve infusing a DNA substitute in proliferating cells and tracking cells as they exit the cell cycle and migrate to their destination (Bayer, 1980). These thymidine analogs are infused in the brain. Accordingly, they are invasive and are only amenable to model systems. Instead, information obtained from invasive methods in model systems is combined with indirect methods to make inferences about the levels of human hippocampal neurogenesis. Doublecortin (DCX) is expressed in recently generated neurons, but not in fully mature neurons, and so is used as a marker to quanitify hippocampal neurogenesis in humans (Fig. 4; (Lee and Thuret, 2018; Sorrells et al., 2018; Charvet and Finlay, 2018; Amrein et al., 2011). Doublecortin is strongly expressed around birth in hippocampal granule cells and declines rapidly during postnatal development. This is true in mice as it is in marmosets. Translating temporal profiles of hippocampal neurogenesis from multiple model systems to humans reveals that human hippocampal neurogenesis should drop sharply during childhood to hard-to-detect levels around adolescence in humans (Sorrells et al., 2018). Examples such as these show the utility of the Translating Time resource to frame whether the tempo of a biological process is unusual relative to those of other species. The inclusion of multiple species is important because it permits identifying conserved temporal trends in hippocampal neurogenesis, and trends that are most likely to apply to humans.

### 3.2. Maturity state at birth

The human neonatal period is a vulnerable phase of transition. Identifying a model system that is similar in degree of maturity at birth to humans would be valuable to treat conditions that arise during birth. We have yet to identify a model system that recapitulates the state of maturity for humans. The state of maturity at birth varies widely across species and along a precocial to altricial spectrum. On the one hand, precocial species are born in an advanced state and after a relatively long gestational period whereas altricial species bear undeveloped offspring born after a relatively brief gestational period. Precocial species can feed themselves, and they can move independently shortly after birth relatively shortly after they are born (Couillard-Despres et al., 2005; Kalusa et al., 2021). In contrast, altricial species are helpless and necessitate substantial parental care for an extensive period (Couillard-Despres et al., 2005; Kalusa et al., 2021). Altricial and precocial species are found across distantly-related vertebrate groups. For example, owls, kangaroos, squirrels, cats, and dogs are altricial whereas ducks, zebras, and seals are precocial (Couillard-Despres et al., 2005; Kalusa et al., 2021; Scheiber et al., 2017; Gómez-Robles and Sherwood, 2016). Humans are considered secondarily altricial because they exhibit altricial and precocial traits at birth, and identifying a suitable model system for humans is still a challenge.

Specifically, humans have relatively long gestations, and have developed mature organs in utero, but they are helpless at birth. That is, hearing is functional by the beginning of the third trimester in utero (Workman et al., 2013; Cusack et al., 2024; Birnholz and Benacerraf, 1983), and eyes are open by birth, which are traits found in precocial species (Workman et al., 2013; Cusack et al., 2024). Humans are also dependent on parental care for an extended period, which is an attribute that is generally found in altricial species. These attributes make humans (and some other species like lemurs) secondarily altricial (i.e., precocial in state of maturity but helpless at birth (Gómez-Robles and Sherwood, 2016; Workman et al., 2013; Cusack et al., 2024). In humans, the state of maturity is similar to those of great apes, suggesting little modification in hominid evolution. Yet, the state of maturity of human newborns is very different from mice.

There are important evolutionary modifications in the timing of birth across mammals, but humans and great apes are similar in degree of maturity at birth. If the relative age of birth were modified in the human lineage, human birth should deviate from other time points when compared to those of great apes, but that is not the case. Instead, birth falls in line with the timing of other biological and behavioral pathways in humans as in chimpanzees (Fig. 3) as well as in gorillas (Fig. 3). We did find that the pace of some locomotor-related anatomical traits deviates in humans relative to great apes, suggesting that the pace of locomotor development has shifted in humans (Blomquist, 2009). The extended development of some locomotor-related traits imply that humans have a relatively extended duration of childhood and helplessness during which infants can absorb vast amounts of information. Learning without the need for immediate task performance results in a powerful foundation model that supports rapid and versatile learning later (Cusack et al., 2024).

The relative timing of birth varies across humans and more distantly related mammals. In mice, birth is relatively advanced compared with humans (Figs. 1 and 3). Mice have one of the shortest gestations of any placental animal in our dataset (i.e., 18.5 days) and rank alongside marsupials which are born in a somewhat fetal state since they continue development within a pouch. In contrast, humans have a long gestation of 270 days, outranking all other primates and ranking alongside large ungulates (e.g., cows) and pinnipeds (e.g., seals), which are precocial groups of animals. Interestingly, the state of maturity at birth in humans is in some ways more similar to sheep than it is to mice (Fig. 3). Considering sheep are very precocial, the similarity in maturity state across humans and sheep at birth exemplify how precocial humans are in terms of their sensory maturation, while they are also helpless for years after birth.

Altricial species like mice have many practical advantages as animal models. Altricial species are born less developed, so it is relatively easy to record developmental time points such as eye opening and walking onset ex utero. Experimental manipulations to study development, such as the total prevention of visual inputs to the brain by sewing the eyes shut before they open, are more easily conducted ex utero. Precociality in mammals is associated with a reproductive strategy which includes small litter size, large body size, and long gestation (Kalusa et al., 2021). These features are not desirable in animal models in laboratories. Yet, they are human features and it would be advantageous to further investigate precocial models to provide a comparative context in which to understand human normal and pathological development, and the evolutionary changes that characterize human life histories.

The human combination of altricial and precocial features at birth is just one example of how humans show a mosaic of features, which they share with different species. As such it is difficult to find a specific ‘best’ model system for all human conditions, but rather it can be beneficial to look at different models depending on the system and conditions.The stage of maturity of humans at the time of birth is important for healthy medical outcomes, and deviations from it can lead to pathologies. Currently, 11.1 % of all live births worldwide are preterm (before 37 weeks of gestation; Birnholz and Benacerraf, 1983). This presents challenges in addressing conditions associated with early birth. The uterine environment is hypoxic compared to the postnatal one so that birth marks a major transition in how oxygen is supplied to the brain (Gopagondanahalli et al., 2016; Dunwoodie, 2009). In full term infants, compared to preterm, there is an extended stage for making the transition, since the uterine environment becomes progressively less hypoxic between the 37th and 40th weeks of gestation (Filippi et al., 2023). Therefore, preterm birth can be associated with conditions related to oxygen intake below or above what is typical in the uterine environment. Several diseases emerge at birth and are associated with the transition in oxygen supply from prenatal to postnatal environments (e. g., retinopathy of prematurity, hypoxic ischemic encephalopathy (Cornet et al., 2023). For example, neonatal hypoxic ischemic encephalopathy (HIE) is an umbrella term for a brain injury that happens before, during, or shortly after birth when oxygen or blood flow to the brain is reduced or stopped and is one of the most common causes of neonatal morbidity and mortality, with a population incidence of 1.7 per 1000 (Lim et al., 2023). Preterm infants are at greater risk of developing intermittent hypoxia due to multiple factors, which includes the immature respiratory systems (Lim et al., 2023; Cornet et al., 2023). Aligning animal models of neonatal hypoxia to human developmental stages remains a challenge (Cornet et al., 2023). Mice do not resemble humans in their state of maturity of birth (Fig. 3). Studying model systems that resemble humans in their state of maturity at birth would be optimal to translate their findings to humans.

## 4. Section III: future directions

### 4.1. Revolutionizing comparative research opportunities with open science

Novel collaborative scientific frameworks, including open science and big team science have much potential to address long-standing issues (Box 1). Increased species diversity coupled with an expansion of open and inclusive science platforms are designed to facilitate interdisciplinary communication (Hamdy et al., 2020). Open science is a movement for better research transparency, sharing, and inclusivity. A major aim of open science is to address the “replication crisis” which is the finding that many published research results have not been reproducible. The open science movement has transformed the way scientists work. It has led to infrastructure such as the Open Science Framework (osf.io), a platform to enable open science practices. The Allen Institute embraced open science values at their core, and focuses on generating and sharing data to enable researchers around the globe to access the extensive and detailed datasets generated for investigation. In order to expand datasets, broader representations, and more inclusive membership, open science has inspired big team science, whereby individual researchers pool resources to take part in large collaborative projects.

Significant progress has been made by the big team science initiatives such as the “Many X” Projects (Hamdy et al., 2020; Coles et al., 2022). This team uses an open science and collaborative approach to enable them to answer research questions with more statistically powerful datasets. It is only possible to robustly apply phylogenetic comparative methods to study the evolution of a biological feature if enough biological units (usually species) are represented. Therefore, large-scale comparative initiatives that seek to include as many species as possible when designing experimental pipelines and recruiting team participation are particularly relevant for increasing the power of cross-species comparison, and to test the interactions of genotype and a phenotype in health and disease.

ManyPrimates focuses on studying a broad range of species to inform the evolution of primate cognitive capacities. These international collaborative initiatives aim to advance the field of primate cognition research and integrate research across a variety of settings including zoos, sanctuaries, laboratories, and field sites. These large-scale, collaborative projects address the limitations currently affecting comparative psychology and primate cognition studies. These collaborative efforts bring together researchers to facilitate collaboration, harmonize data, and overcome issues that are typical of primate behavioral studies. Challenges include poorly preserved and biased samples, and varying methodologies employed by different researchers, coupled with a limited infrastructure to integrate findings across human and primate data. This new approach overcomes many existing past challenges that were pervasive in primate behavioral studies. Projects in the Big Team Science Network under the umbrella of Many X (e.g., ManyPrimates, ManyManys, ManyZoos, ManyGoats, ManyBabies) have developed new workflows to integrate information across sites, improve performance, and sample size. Together, big team science and open science have brought on new infrastructure with which to do science.

### 4.2. A one health model system broadens approaches and species

**Fig. 5.**
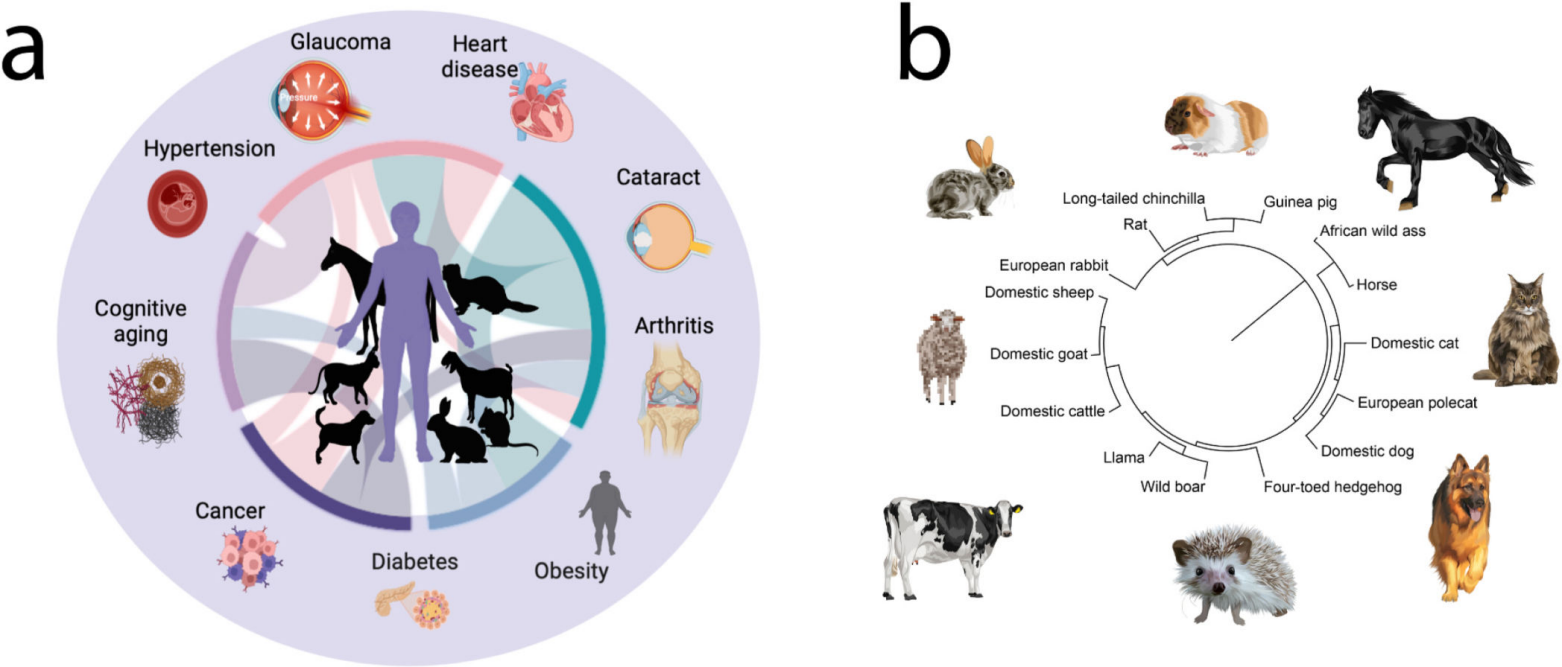
(a) Many health conditions found in humans are also found in other species. Those include obesity, cancer, and cognitive aging. (b) We have included a phylogeny of some species seen in veterinary clinics. The integration of human and veterinary medicine could help tackle many chronic diseases found in humans. The phylogeny in (b) is generated with phyloT.

There are many ways to include diverse species in research. Although we traditionally consider model systems as laboratory animals that are experimented on, there are additional approaches that could be employed to address problems in biomedical sciences (Brenowitz and Zakon, 2015; Many Primates et al., 2019). Much progress could be made if model systems were studied through the lens of the “One Health” perspective, which posits that enhancing animal and human health can synergistically improve both human and animal health (Atlas, 2013; Natterson-Horowitz, 2015). Concentrating on model systems that naturally manifest diseases relevant to humans and those that are already treated in the veterinary clinic has the potential to advance medicine (Atlas, 2013; Natterson-Horowitz, 2015; Landsberg et al., 2015; Youssef et al., 2016) Yet, resources from veterinary medicine are rarely considered in biomedical sciences (de Sousa et al., 2023; Gould et al., 2015). Much of biomedical sciences relies on a handful of model systems, and especially mice even though mice rarely recapitulate many of the complex human diseases (e.g., arthritis, cataracts, or Alzheimer -like disease) we seek to study (Fig. 5). To address this limitation, mice are genetically engineered to mimic specific aspects of human diseases. Even though genetic modifications have improved with CRISPR-Cas9 systems (Ishibashi et al., 2020; Yang et al., 2014), these genetically modified mice usually only partially represent the complexities of human conditions (Bilkei-Gorzo, 2014; Onos et al., 2016), limiting our understanding and the translatability of their findings to human health (Brenowitz and Zakon, 2015). Moreover, traditional animal experimentation conducted in confined environments and controlled laboratory settings does not reflect the conditions in which they develop in humans.

A contemporary, ethically driven approach should advocate to study shared environments to allow animals to recapitulate their natural behaviors in health and in disease. This holistic tactic supports the principles of One Health, which advances the notion that what’s beneficial for the health of animals can be advantageous for humans. In other words, enhancing animal and human health to study biomedical sciences can yield invaluable insights into the health of model systems and humans. We can gain a clearer understanding of the efficacy and safety of pharmaceutical interventions, significantly enhance the success rate of clinical trials, reduce animal suffering, and expedite life-saving treatments by integrating approaches that seek to improve the health of humans and animals. Yet, there has been little coordinated effort to leverage these spontaneous model systems for the mutual benefit of human and animal health.

Companion animals develop diseases that resemble those found in humans (Narfström et al., 2013; Youssef et al., 2016). Domestic animals include household companion animals (e.g., cats, dogs) as well as livestock (e.g., cows, goats). These animals share our lifestyle and our environment and develop many diseases found in humans. These companion animals spontaneously develop complex conditions such as cognitive dysfunction (akin to dementia), cardiomyopathies, cataracts, and arthritis (Landsberg et al., 2012; Narfström et al., 2013; Youssef et al., 2016). Such overlap in disease incidence warrants the creation of a new kind of model system, which we refer to as a One Health model system. In contrast to the traditional model system, a One Health model system is studied to enhance the animal’s health as well as our own, and how their health interacts with a sustainable environment. Non-invasive studies combined with targeted treatments in companion animals could be useful approaches in biomedical sciences. There is significant potential in studying these animals and conducting clinical trials to enhance our own health. Moreover, such an approach alleviates issues linked to the ethical use of animals in research because it strives to replace experimental systems, and instead focuses on the health of the animal, and the environment. This One Health model system is applicable to many diseases because companion animals and humans suffer from overlaping diseases s (Bernard et al., 2024; Thomas et al., 2020). For example, humans and dogs can develop cancers (e.g., osteosarcoma, bladder cancer), inflammatory bowel disease, epilepsy, chronic kidney disease, and cognitive dysfunction syndrome. The extent of inbreeding and artificial breeding should be factors for consideration when selecting a model system for humans. Some breeds may vary in their suitability to study human disease because they are at increased risk of diseases resulting from artificial breeding (Thomas et al., 2020; Bannasch et al., 2021; Cecchi et al., 2020; Estevam et al., 2022; Malik, 2001). Among possible candidates, cats are of particular interest for study because most cats are domestic shorthair and have not been subject to intense human breeding compared with other animals used as companions (e.g., purebred dogs) or as livestock (e.g., cows, chickens). A One Health approach that seeks to understand human diseases, as well those of companion animals (Bernard et al., 2024) is a sustainable approach to improve biodiversity.

## 5. Conclusion

We have reviewed multiple cases where the study of diverse species can help address long-standing biomedical sciences (Box1). Historically, neuroscience drew vitality from the study of diverse species. Although mice have been useful to advance many issues in biomedical sciences, it is now time to reintroduce diverse model systems in neuroscience. The Translating Time approach focuses on comparing developmental time points to identify conserved and derived traits across a broad range of species. Shifts in the timing of events in the human lifespan have shown great potential to explain human specific features; by calibrating these differences to a common event scale, we can connect biomedical knowledge about humans to other species. This tool has much potential to integrate multiple kinds of information. Future endeavors could connect human health and the health of endangered animals for a sustainable approach to improve health and biodiversity.

## Supplementary Material

1

## Data Availability

No data was used for the research described in the article.

## Box 1 Future Directions and outstanding questions

1. Complimentary model systems: The development of complimentary model systems could be useful to enhance human health. How could we achieve this, and what should we focus on?
2. Inclusive approaches in science: How can we create inclusive environments in science for model systems and humans?
3. Translational sciences: How can the insights gained from studying developmental processes be translated into medical applications. How can these findings more effectively contribute to the development of new therapies, treatments, or diagnostic tools for age-related diseases?
4. Community engagement: There is a need to involve the community in biomedical sciences. Community is broad and includes students, educators, and the general public. What strategies can be utilized to attract the public to develop the species database, incorporate new types of data, update science platforms, and collaborate with emerging technologies?
