# Supplementary material for: Translating time: Challenges, progress, and future directions
Source: Brain Res Bull. Author manuscript; Available in PMC 2025 Mar 13. (PMC11904871; doi:10.1016/j.brainresbull.2025.111212)
Supplement: 1 [file NIHMS2055635-supplement-1.docx]

**Supplementary material**


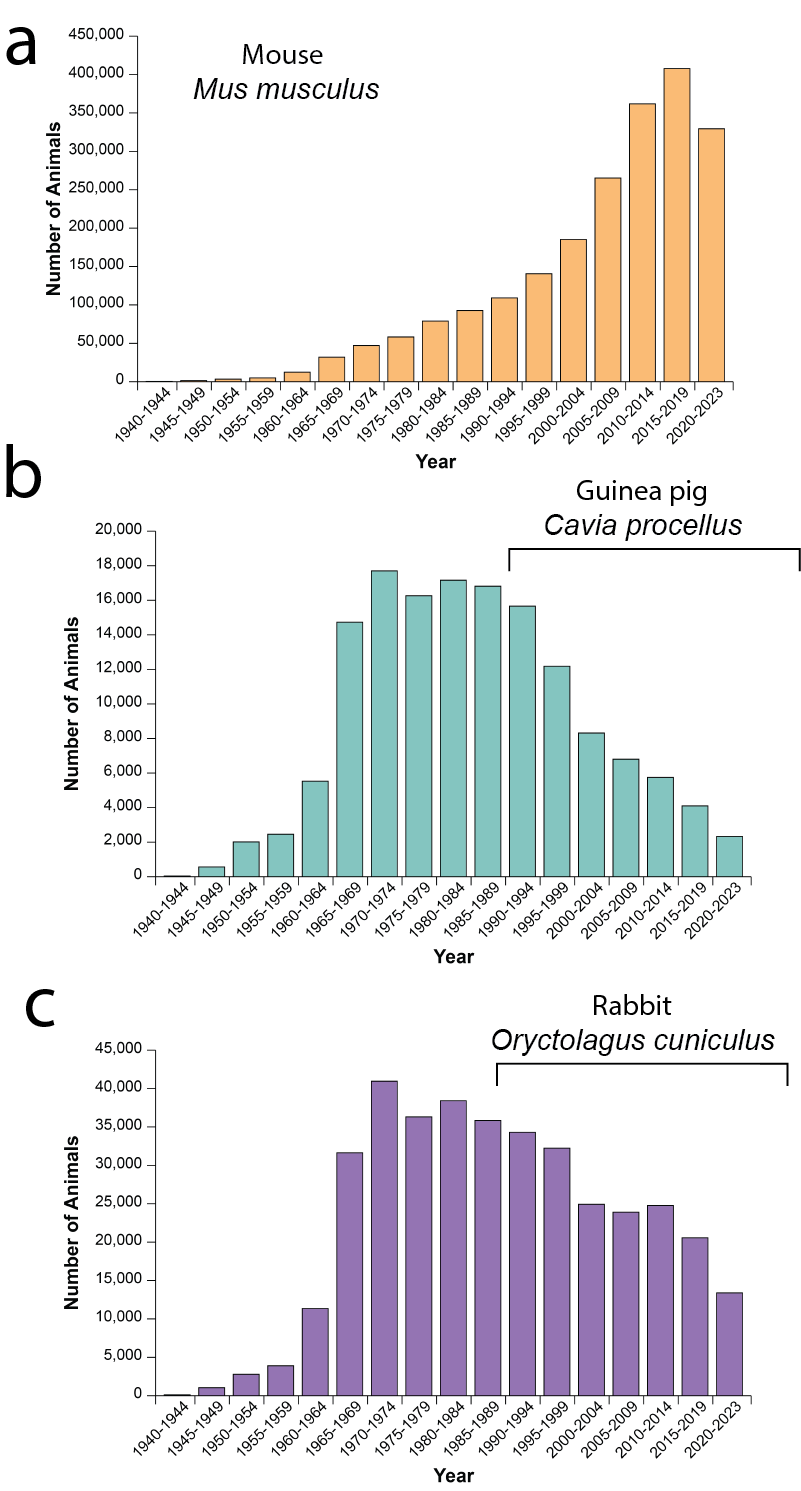


**Supplementary Fig. 1.** Number of studies focused on different groups of species, including mice (a), guinea pigs (b), and rabbits (c). We used the key word of the species name and downloaded the number of studies reporting on each study. The number of studies reporting on guinea pigs (b) and rabbits (c) declined since the 1990s, but not for mice. Data is from pubmed and data for each year was averaged across 5 year bins.


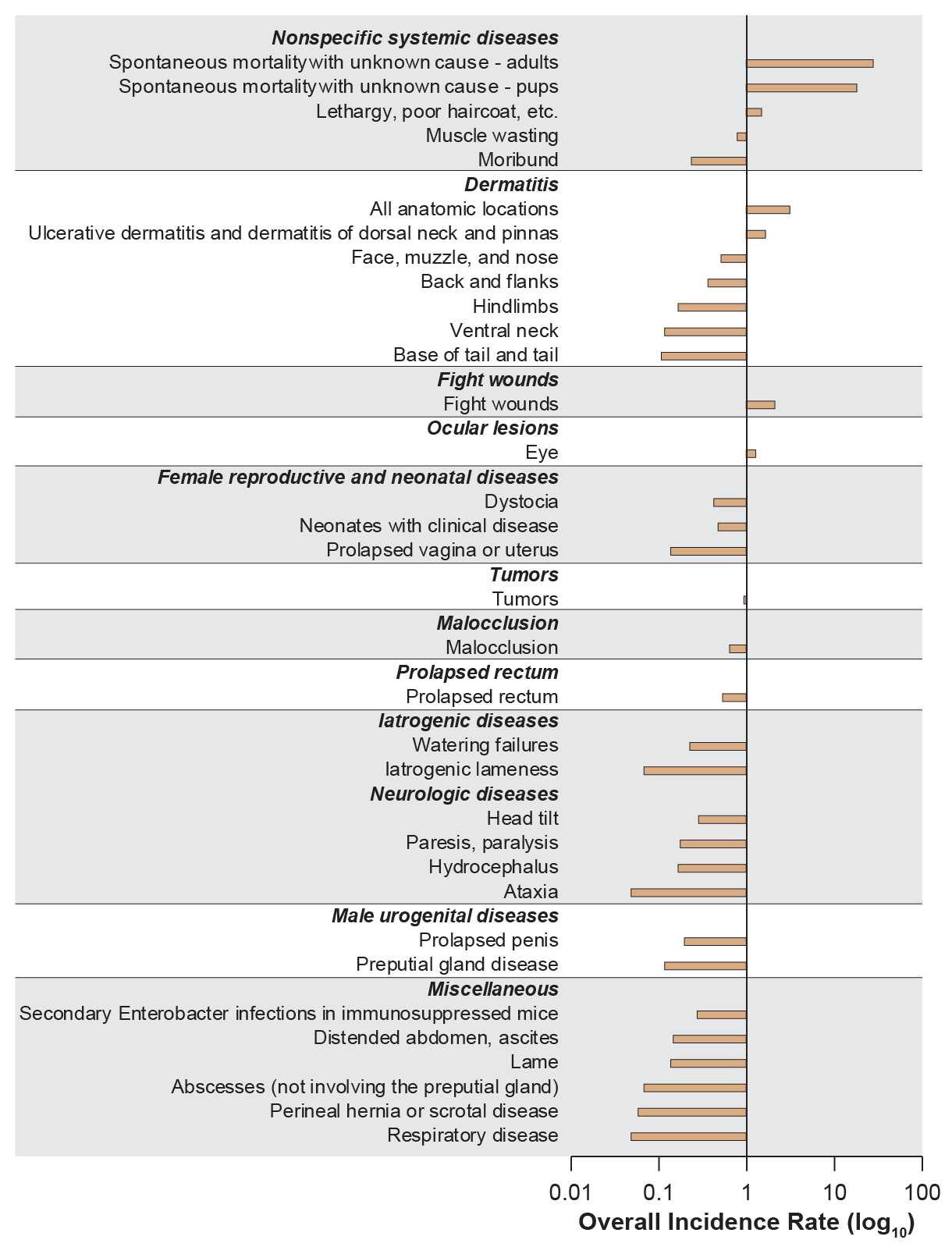


**Supplementary Fig. 2.** Summary of disease incidence impacting laboratory mice across multiple facilities. These data are from Marx et al. [23].
